# Supplementary figures and images for: Genomic and morphological evidence of distinct populations in the endemic common (weedy) seadragon Phyllopteryx taeniolatus (Syngnathidae) along the east coast of Australia
Source: PLoS One. 2020 Dec 23;15(12):e0243446. doi: 10.1371/journal.pone.0243446 (PMC7757807; doi:10.1371/journal.pone.0243446)

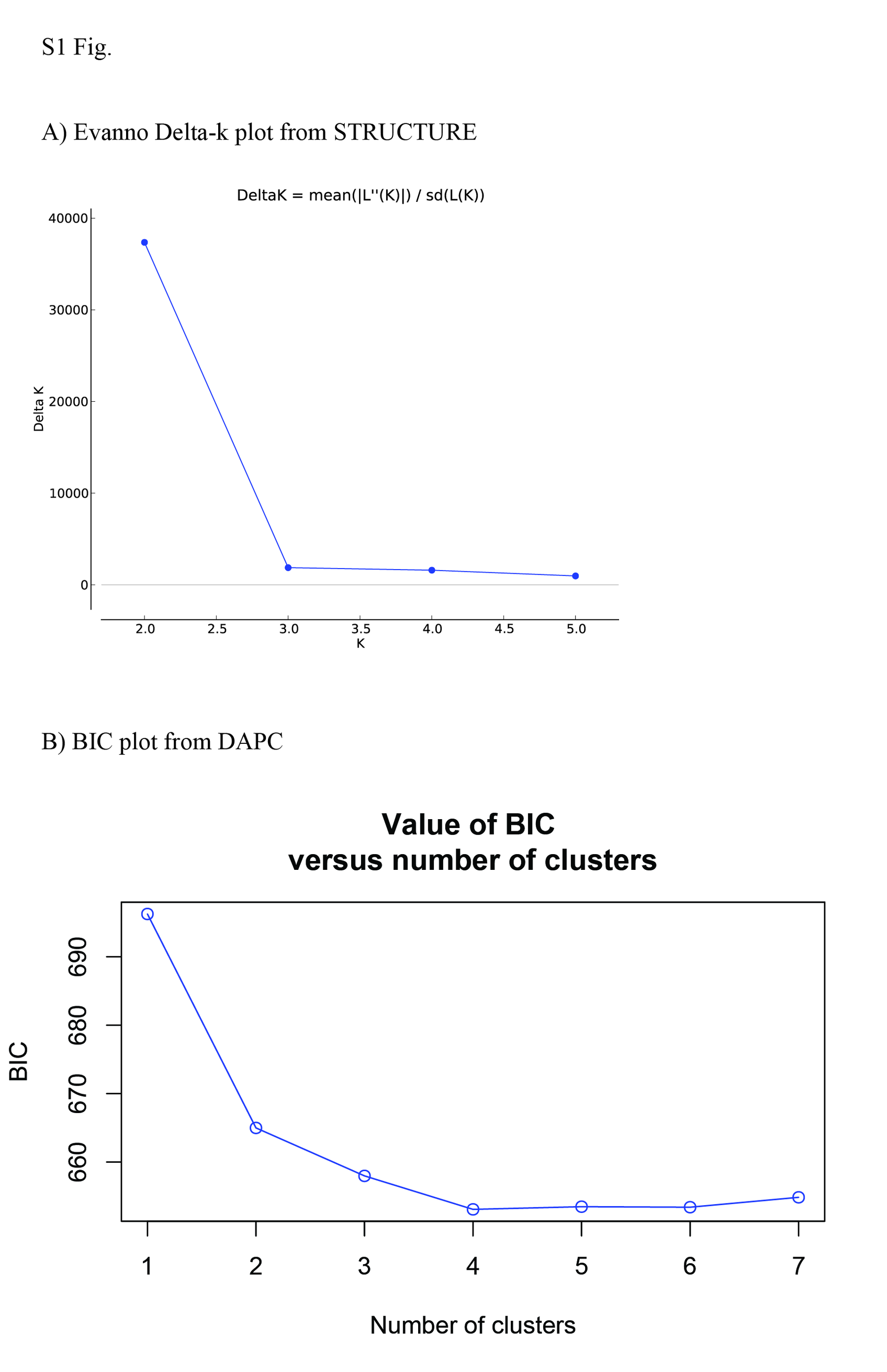

Supplement: S1 Fig — (A) Evanno delta-K plot. Results from STRUCTURE HARVESTER run on 72 individuals of the seadragon P. taeniolatus from southeastern Australia. (B) Bayesian Information Criterion (BIC) plot. Results from find.clusters() method run in ADEGENET on 72 individuals of the seadragon from southeastern Australia. BIC value decreases until k = 4, then begins to slightly increase at k = 5, indicating 4 is the most likely number of clusters. (TIF) [file pone.0243446.s001.tif]

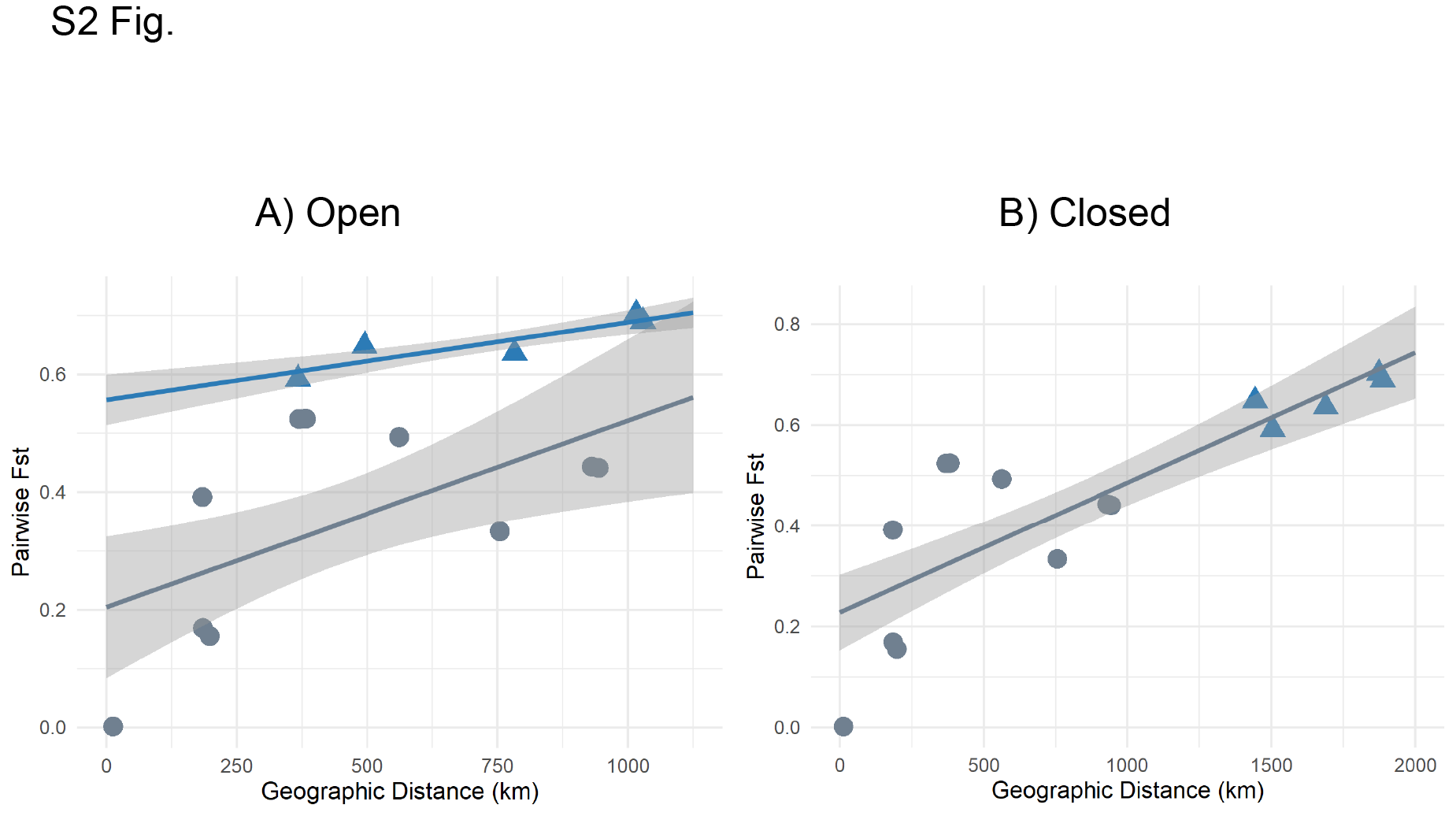

Supplement: S2 Fig — IBD results for (a) open connection (no land bridge) and (b) closed connection (Bassian Isthmus historical land bridge). Blue triangles are pairwise comparisons including Victorian populations, grey circles are all other pairs. (TIF) [file pone.0243446.s002.tif]

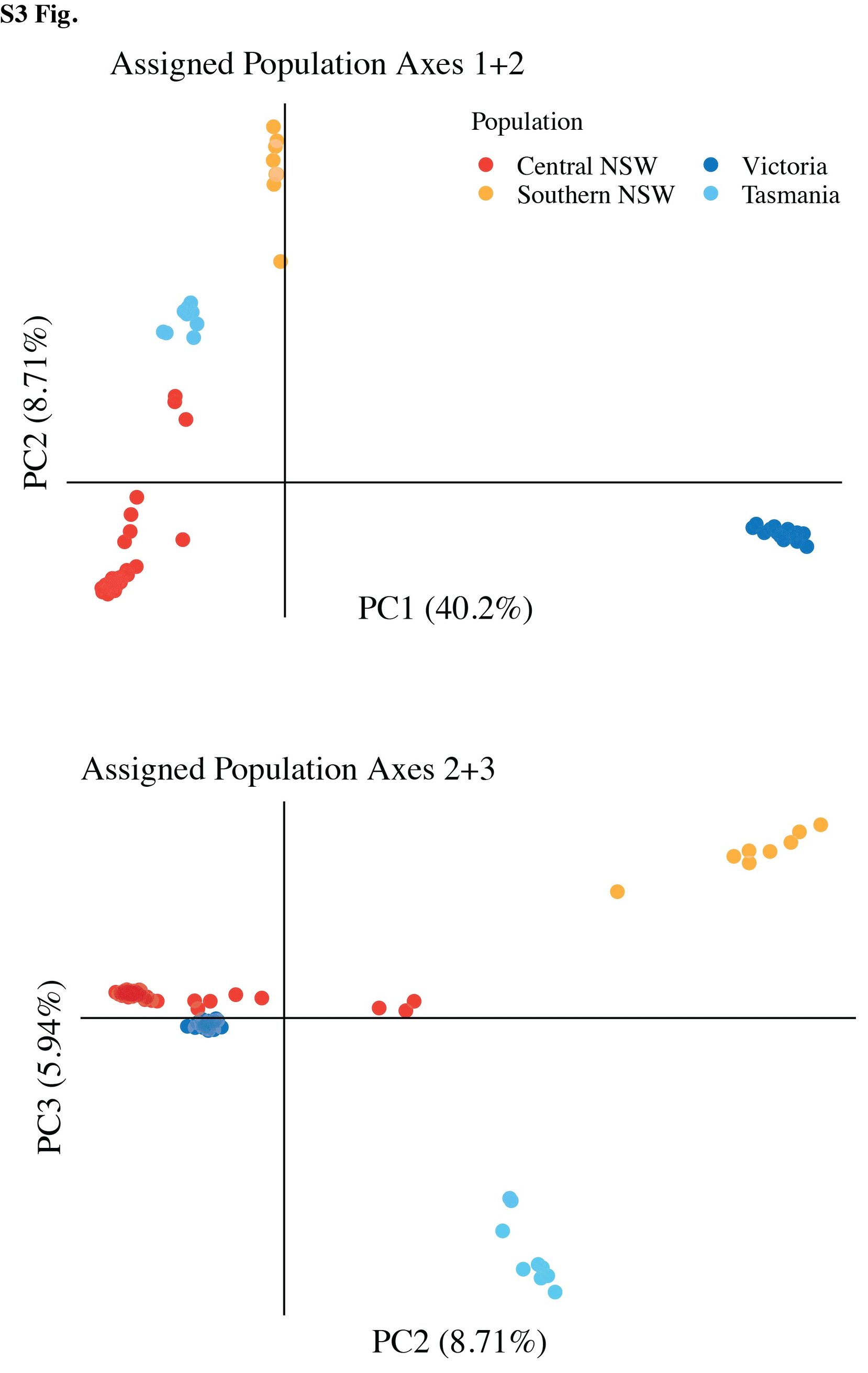

Supplement: S3 Fig — (A) Results of PC1 and PC2 and (B) PC2 and PC3 were run on 72 individuals of seadragon from southeastern Australia using the R package ade4. (TIF) [file pone.0243446.s003.tif]
