# Supplementary material for: Genomic and morphological evidence of distinct populations in the endemic common (weedy) seadragon Phyllopteryx taeniolatus (Syngnathidae) along the east coast of Australia
Source: PLoS One. 2020 Dec 23;15(12):e0243446. doi: 10.1371/journal.pone.0243446 (PMC7757807; doi:10.1371/journal.pone.0243446)
Supplement: S3 Table — AMOVA of (A) all nine P. taeniolatus populations, and (B) grouped into four populations (CNSW, SNSW, VIC and TAS). (DOCX) [file pone.0243446.s007.docx]

S3 Table AMOVA results.

(A) AMOVA of all 72 individuals grouped into 9 populations* as per Fig 1 Map.

| Source of variation | d.f. | SS | % Variation | *F*-value | *p* |
| --- | --- | --- | --- | --- | --- |
| Among populations | 8 | 76826.493 | 56.16 | *F_ST_* = 0.562 | 0.0001 |
| Within populations | 135 | 63398.59 | 43.84 |  |  |

* Nine Populations: Botany Bay, Sydney, Jervis Bay/Guerilla Bay, Wollongong, Eden (all NSW); Flinders, Portsea (both VIC); Bicheno, Hobart (both TAS).

(B) AMOVA for all individuals, grouped into four subpopulations based on STRUCTURE.

| Source of variation | d.f. | SS | % Variation | *F*-value | *p* |
| --- | --- | --- | --- | --- | --- |
| Among groups* | 3 | 70657.046 | 58.75 | *F_CT_* = 0.587 | 0.0004 |
| Among population within groups | 5 | 6169.448 | 4.24 | *F_SC_* = 0.103 | 0.0001 |
| Within populations | 135 | 63398.59 | 37.01 | *F_ST_* = 0.630 | 0.0001 |

* Four subpopulations: CNSW - Central New South Wales, SNSW - Southern NSW, VIC - Victoria and TAS – Tasmania.
