# Supplementary material for: Genomic and morphological evidence of distinct populations in the endemic common (weedy) seadragon Phyllopteryx taeniolatus (Syngnathidae) along the east coast of Australia
Source: PLoS One. 2020 Dec 23;15(12):e0243446. doi: 10.1371/journal.pone.0243446 (PMC7757807; doi:10.1371/journal.pone.0243446)
Supplement: Attachment — Submitted filename: Common_seadragon review.pdf [file pone.0243446.s011.pdf]

Klanten et al. have done a careful job at addressing the reviewers' concerns and have added useful figures and additional material that improve the manuscript. My remaining concerns are regarding the still strong emphasis on a species complex, for which I do not see sufficient evidence. There are also questions regarding the PCA, which I think is a simple plotting error.

1) Both reviewer 1 and myself have raised concerns about the suggestion of a species complex in Victoria and New South Wales. The authors have made modifications in calling for extended sampling and the improved resolution that nuclear markers can give, which are useful additions. However, the text still strongly implies that there is a species complex but that separate species are "not yet advocated". The presented evidence based on allele frequency differences analyzed in a number of ways is not strong enough to draw that conclusion. This needs justification from the literature. As I have pointed out in my last review, this view is omitting the results from Structure at K=2 that does show shared ancestry components between Victoria and the eastern sites. If there was reproductive isolation, one would expect completely separate ancestry proportions. The Structure results need to be mentioned.

2) Along the same vein, there are also multiple mentions of "very restricted to no gene flow" or similar. This lies on the assumption that FST values reflect migration rates. In practice, this relationship is not well established because there are also other factors which impact allele frequency differences (see for example McCauley DE, Whitlock MC. "Indirect measures of gene flow and migration: $FST \neq 1/(4Nm + 1)$" *Heredity*. 1999;82:117–125). It would be more appropriate to refer to "significant allele frequency differences" or "significant genetic differentiation", because this is what has actually been measured with FST.

3) I thank the authors for including the PCA analysis. I think there may have been a mistake in the PCA plotting. I wondered how such clear clustering in DAPC can result in the PCA plot as shown in Figure S3, where several individuals were not clustering with their parent population. If that was true this would be concerning because PCA and DAPC are conceptually very similar. I repeated the analysis (on genepop file with four population groups separated) and found a clear assignment of individuals to populations (see code and plot below). The structure of the PCA looks similar to Figure S3 but it looks like the coloring of the individuals according to their populations is off in Figure S3. There must be a glitch in the R script that the authors used. Good news is that the PCA will support the DAPC results much better and the authors could consider to include it in the main text (up to the authors).
(The screenshots will likely not appear in the online form, so please see the attached PDF version of the reviewer comments)

```
library(adegenet)
library(ade4)
library(ggplot2)

t <- read.genepop("~/Downloads/S2 Data.gen")

levels(t$pop) <- c("CNSW", "SNSW", "VIC", "TAS")

X <- scaleGen(t, NA.method="mean")
pca1 <-  dudi.pca(X, cent=F, scale=T, scannf=F, nf=2)
pl <- ggplot(data=pca1$li, aes(x=Axis1, y=Axis2, color=t@pop, label=row.names(pca1$li)))
pl + geom_point() + scale_color_brewer(palette="Paired")
```

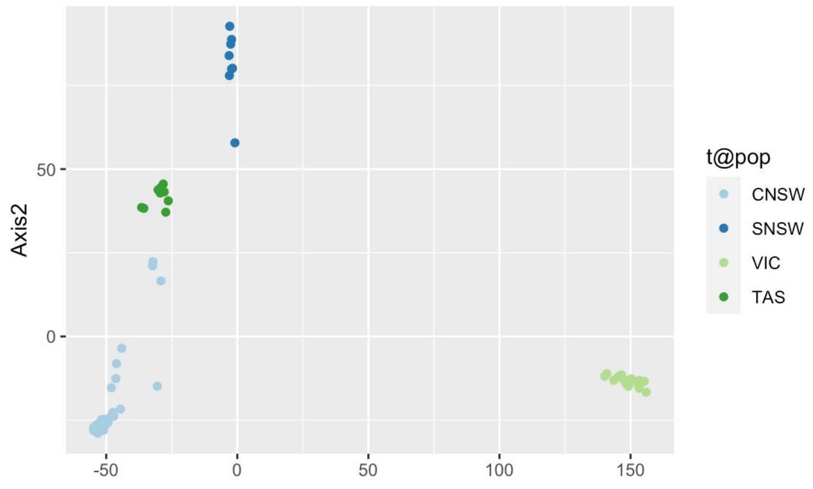

Minor comments

L.67 Not sure if reference 19 is appropriate for the number of Syngnathidae species; Reference 18 Eschmeyer actually lists 321 valid species

L.129ff. May be more readable as complete sentences?

L.219 Typo in “Principal components”

L.230 Typo in “patterns”

L.231 Population code “SYD” has not been defined before.

L.258 Unclear what the difference between FST = 0.561 and FST = 0.630 (L.262) is.

L.289 Move “(FST ranging 0.636– 0.703)” to the end of the sentence to include TAS

L.313 Reference to Supplementary Figure S1 needed.

L.370 Past tense? “were highly distinct”

L.373 Move references to after “as a barrier to marine dispersal for 20-25 kya
374 during the last glacial maximum (LGM)”

L.376 The strait opened 14 ka ago according to Lambeck & Chappell, so gene flow may have been reinstated between 14 kya and today, not 14-25 kya.

L.379 Type in “shape differences”

L.380 It does not seem obvious why sex differences would not affect the observed differences.

L.383 It is more appropriate to refer to allele frequency differences than gene flow (see above).

L.386 Need a reference that the found differentiation is extraordinarily high.

L.388 This indicates that some of the RAD loci obtained the mitochondrial genome. Is this likely? Intuitively, I would think that is unlikely to obtain mitochondrial RAD loci but maybe this is different in this case. If there were mitochondrial loci in the dataset, these would have to get filtered or modeled separately from nuclear loci because they are haploid and have a different effective population size.

L.398 This paragraph seems isolated and the point is not clear.

L.405 Unclear which populations "this division" refers to. The previous sentence states that TAS and NSW populations were historically connected, not divided. The following sentence also talks about similarities, rather than divisions.

L.415 change to "(see also [30])"?

L.435 Reference needed: "less resilient under normal circumstances, but instead have a smaller gene pool due to smaller population sizes and restricted distribution ranges"

L.436 It is more appropriate to refer to allele frequency differences than gene flow (see above).

L.440 Reference needed: "increase in anthropogenic stressors resulting in habitat loss (kelp and seagrass) and an increase of sea surface temperature"

L.458 It is more appropriate to refer to allele frequency differences than gene flow (see above).
