# Supplementary material for: Genomic and morphological evidence of distinct populations in the endemic common (weedy) seadragon Phyllopteryx taeniolatus (Syngnathidae) along the east coast of Australia
Source: PLoS One. 2020 Dec 23;15(12):e0243446. doi: 10.1371/journal.pone.0243446 (PMC7757807; doi:10.1371/journal.pone.0243446)
Supplement: Attachment — Submitted filename: Response to Reviewers.docx [file pone.0243446.s010.docx]

Response to Reviewers:

Manuscript: **PONE-D-20-17523**

Genomic and morphological evidence of distinct subpopulations in the endemic weedy seadragon *Phyllopteryx taeniolatus* (Syngnathidae) along the east coast of Australia

**Reviewer #1:**

Summary

Klanten et al., present a population genetics study on the weedy seadragon (Phyllopteryx taeniolatus) using SNP sequence data generated from ddRADseq. This work builds on previous studies that used mitochondrial and microsatellite sequence data. In this study the authors have contributed further evidence for significant population structuring at a regional scale that has implications for the management of this species. They have also incorporated morphological measurements as further evidence of population isolation and interpret these results along with the genetic data with reference to the historical Bassian Isthmus as a barrier to dispersal.

The SNP data generated in this study have been assembled and filtered appropriately to produce a high-quality dataset for which to analyse genetic diversity and patterns among the populations. The authors have used multiple different analyses to test for genetic population structure, and these have been carried out appropriately for the dataset. The phylogenetic analyses are not suitable for this data set however, and should be removed (comments below). There is also widespread unjustifiable use of the word hybridisation, which needs to be removed. Otherwise, this is a robust contribution to understanding these iconic fish.

**Response:** We thank Reviewer 1 for their constructive comments. We accept the reviewers point about the phylogenetic tree (Figure 5) and have opted to remove this from our manuscript. It is unnecessary as our conclusions are well supported by the remaining analyses.

We also edited the word hybridisation using terms such as ‘interbreeding’

Note: We added additional citations (suggested by reviewers) so the citation # in our manuscript have changed.

**Specific comments:**

1. Line 59: “…(taxonomically known as the common seadragon)” isn’t correct as common names are not controlled by the ICZN. Suggest change to: (also known as the common…).

**Response**: Edited as suggested by reviewer. We also mention (Title and Abstract) both “common or weedy”.

2. Line 62: Ruby seadragons are thought to have prehensile tails (Rouse et al. 2017 First live records of….Mar Bio Records).

**Response**: Thank you for pointing this out, we changed the sentence to make sure it’s only weedy seadragons that have no prehensile tails.

“*Phyllopteryx taeniolatus* are weak swimmers and lack the prehensile tail of seahorses ...”

We also added Rouse et al. 2007 [22] as a reference.

3. Lines 77-88: You state that mtDNA showed strong population structure for weedy seadragons, and lowered genetic diversity on the east coast. Yet at the end of this paragraph you state that leafy seadragon genetic diversity was moderate to low, a similar pattern to the weedy. This last statement is in contrast with [28] which actually reports some areas with quite high diversity, but showed low diversity for the east coast. You either need to elaborate further on this complexity, or just shorten the final sentence of the paragraph so it does not include a species comparison.

**Response**: We edited the sentence so that it does not include a comparison.

“Another study [34] on the leafy seadragon (*Phycodurus* *eques*), using both mtDNA and microsatellites, also found strong population structure especially between western and southern Australian populations, with high to low genetic diversity.”

4. Line 149: Were pairwise Fst values also calculated in Arlequin or using R? This is not explicitly stated in the Methods section but is stated in the caption to Table 1.

**Response**: We added “… in Arlequin ..” to clarify sentence.

5. Line 155: EAC has not been mentioned in text before here, though it is explained in the Discussion (line 312), I would write it out in full here as well.

**Response**: Eastern Australian Current (EAC) now written in full.

6. Have you corrected for different sample sizes among populations when calculating expected Heterozygosity?

**Response**: We used Nei’s calculation of heterozygosity using the R package pegas. Nei’s equation is as follows: $\hat{H}=\frac{n}{n-1}(1-\sum_{i=1}^{k} p_{i}^{2})$

This calculation does not correct for different sample sizes. Without this correction values calculated from small samples sizes are likely to be biased upward. More to the point we don’t emphasise heterozygosity comparisons between populations and only consider the global (overall) values as they relate to other species.

7. It is not clear exactly how the geographic distances were obtained, using the Ruler tool, by hand in Google Earth, or calculating the edge length of a shapefile? Would this be easy to replicate if it has been measured by hand?

**Response**: we measured by hand using Ruler tool ‘Path’ in Google Earth.

“… using Google Earth following the Eastern Australian Current (EAC) along the east coast to Tasmania using the Ruler tool Path by hand.”

8. Can you display the Evanno delta-K plot from STRUCTURE, and the BIC plot from DAPC that you used to determine the number of appropriate population clusters (K=4). These plots could be added to the Supplementary Material.

**Response**: Plots have now been added to Supporting information (S1 Fig A and B).

9. Line 238: “Our unrooted phylogeny based on Maximum likelihood inferences supports the structure analyses (K= 4) distinguishing populations between CNSW, SNSW, VIC and TAS (Figure 5).” Actually, your unrooted tree shows three clusters and a grade. You may wish to use midpoint rooting to assist with interpretation here. It’s hard to see what is going on with this figure. Having said that, I do not think that its valid to apply this evolutionary model to a SNP data set. RAxML is definitely not designed with SNP data in mind, and you would be better off using software that deals with the assumptions created by this type of data set. If you can’t do this, I would suggest removing this part altogether. It doesn’t really add anything unique to your conclusions.

**Response**: We removed any text referring to phylogenetic tree and Figure 5 as suggested above from our manuscript.

10. Line 238: You state that the “Eden population in southern NSW is a monophyletic clade within the broader NSW group, instead of its own lineage (Figure 5).” This does not make grammatical sense with regard to monophyly, and needs clarification.

**Response**: This sentence has been removed, as we removed all text relating to phylogenetic tree and Figure 5.

11. Line 245: “These results would indicate possible hybridisation between CNSW, SNSW and TAS but not VIC (see K=4, Figure 3).” Firstly, this seems more like Discussion than results, and needs to move to that section. Hybridisation is not the only source of genetic similarity, so do check your assumptions here, and expand on all possibilities unless you can test amongst them. Having said that, hybridisation is not the right term to apply to interbreeding among populations.

**Response**: Removed this sentence from results section.

12. Line 271: “Furthermore, the smaller-sized seadragons in VIC also appear to have a darker colour pattern compared to NSW and TAS seadragons (see Figure 1).” I don’t think you should refer to colour based on the single images displayed, and those that lack a colour standard. There are so many uncontrolled variables here.

**Response**: We removed this sentence. Furthermore, the whole section on morphology has changed as per Reviewer 2.

13. Lines 278-280: Think about removing phylogenetic inference, as suggested above.

**Response**: Removed from manuscript.

14. Lines 286-288: You may wish to also cite [28] here, as it also showed that the Portsea/Flinders animals were distinct from NSW and TAS.

**Response**: We added citation Wilson et al. 2017 [30].

“Of note is that all individuals from two sites in Victoria (Flinders Bay and Portsea) are highly distinct, genetically and to a degree morphologically, to the main populations of central and southern NSW, as well as Tasmania [see also 30].”

15. Line 288-290: “The Bassian Isthmus [29] which has acted as a barrier to marine dispersal for 20-25 kya during the last glacial maximum may have prevented mixing between these populations, during which Victorian weedy seadragons diverged in isolation.” I would acknowledge that this has already been suggested, which you state in your introduction, but do not link to here.

**Response**: We added Wilson et al 2017 [30] in citation to this sentence.

“The Bassian Isthmus [30–32] which has acted as a barrier to marine dispersal …”

16. Line 292: “been ample opportunity for hybridisation”. Do you mean ample opportunity for secondary contact? You don’t usually here about populations hybridising, rather different species hybridising.

**Response:** changed and used gene flow

“Since the opening of the Bassian Isthmus, approximately 14-25 kya, there has been ample opportunity for gene flow, therefore the question arises …”

17. Line 297-298: “we suggest the Victorian P. taeniolatus to be a subpopulation, alternatively weedy seadragons along southeast coast of Australia are forming a species complex [5,48].” It has already been demonstrated that, mitochondrially, the VIC animals are not the closest relatives of the east coast populations, so your interpretations here are somewhat skewed by the fact your sampling gaps increase the differences you see. You really have very little evidence for suggesting a species complex, and I suggest you remove this speculation from here and line 316-320. You may say it’s possible, but you have no evidence for this at present. Also, it would be prudent to refer to the study which has sampled them across the distributional range and did not find evidence for this!

**Response**: We have changed this section and the next and hypothesis that by extending the study in future to include individuals from the entire distribution range and using genome-wide SNPs, this study has increased the power of analysis both by including a large number of markers, and by having markers in more biologically relevant regions of the genome. We would like to point out that mitochondrial data frequently has a different gene tree from nuclear data and is a tiny subset of the genes available for divergence. In addition, to having many more loci (both mt & nDNA), nuclear markers provide a better picture of divergence because they both code for more proteins, and are not only matrilinear, but are less affected by introgression and diverge faster.

We agree that this study alone does not provide sufficient evidence that they are different species, but our populations (and particularly Victoria) are quite divergent with F_ST_ values

well into what separates species in many other organisms. This in fact is important for future management and conservation.

Please see page 14 in MS where we elaborate on this issue.

18. Line 303: you should remove discussion about colour differences as it was not tested here.

**Response**: We removed this section.

19. Line 309: issues with ‘hybridisation’ as discussed above. You just mean interbreeding among populations, which is the foundation of the biological species concept.

**Response**: Changed to ‘.. interbreeding .. ‘

20. Lines 321 – 333: In the first sentence of this paragraph you state the difficulty in comparing the genetic diversity of the SNP dataset to other studies on syngnathids which have used microsatellite sequence data. As the number of alleles does have an effect on expected Heterozygosity values I would urge caution in directly comparing He values between different species and studies where the number of alleles may differ, particularly

when the total number of alleles for this study is likely to be much higher given that thousands of SNP loci were used.

**Response**: We agree and have now added this statement to the end of that paragraph

“However, these data should be interpreted with caution. Theoretically, the maximum heterozygosity derived from biallelic loci (such as the SNPs employed here) is 0.5. On the other hand, heterozygosity at microsatellite loci can theoretically reach 1.0.”

21. Line 358: Again, remove terminology ‘hybrids’

**Response**: Removed and replaced with “interbreeding”

22. Line 367: make ‘scientist’ plural

**Response**: Corrected

23. References- many do not italicize species names. this needs to be fixed.

**Response**: Fixed all references, thank you for pointing out.

**Reviewer #2**

Main points

Species complex:

The authors suggest in the discussion that Victorian and New South Wales populations may be reproductively isolated to form a species complex. This conclusion is in my opinion not supported by the data. As I outline below, the morphological differences need further investigation. The genetic differentiation between those populations is large as per FST but that per se does not imply complete reproductive isolation. The Structure analyses at K=2 indicate some potential shared ancestry components. For such a far reaching claim, one would need additional analyses explicitly showing the absence of gene flow and, as the authors acknowledge, ideally more than one sampling site in Victoria and from the remaining distribution. The conclusion that Victorian populations should be separately managed should therefore be carefully considered. I also wonder what the recommendation would be about Tasmanian and New South Wales populations. According to the given scenario. they were possibly historically connected when the Bassian Strait was closed but now they are separated. What would be the recommendation for their management given that their differentiation towards New South Wales is smaller?

**Response**: We thank Reviewer 2 for their constructive comments.

As Reviewer 1 (point 17 above) also pointed to this issue, we clarified that our data has limited evidence, for the moment, for a species complex, and we suggest that much more sampling (across its entire range) using genome-wide SNPs will be needed. We would like to leave this hypothesis in our manuscript as a possibility (assumption) that it may occur. It will open dialogue and direct to more studies into this species. We have elaborated on this extensively in our discussion see p.14 in MS (also see our extensive response to Reviewer 1 above).

Regarding management we are suggesting that each state should manage their populations in particular VIC.

Morphological analysis:

I find the conclusion that the New South Wales and Victorian populations are morphologically distinct premature. The main difference is a difference in body size. A difference in body size could reflect temperature differences or differences in demographics or age structure of the population or even just sampling younger individuals in Victoria. These possibilities should be at least carefully discussed.

We can expect that all other metrics scale allometrically with body size, hence it is not surprising to find further differences between the two populations. The analyses should be performed with measurements that were scaled by the body size.

It is further not clear if the study separated males and females, which do show sexual dimorphism at least in the body depth. It would be great to test here if there are other differences between males and females.

The discussion compares Tasmanian to New South Wales individuals to support evolutionary scenarios but this data was not included in the main analysis. The Tasmanian individuals either need to be included in the main analysis or this discussion needs to be explicit that it is speculative.

Lastly, comparing only New South Wales and Victoria may miss variation that is being seen in the genetics. I would be interested to see a PCA of the morphological measurements of all individuals including Tasmania. It would be interesting to see if morphological structure follows genetic structure.

**Response**: We have now analysed morphological differences via examination of external metrics vs total length (biplots with linear trendlines, see S4 Fig) and via ordination LDA and PCA (Fig 5 A and B). We removed Table 3 and re-wrote the morphology sections in Methods and Results.

The biplots suggest that several metrics differ between Victorian individuals and both NSW and Tasmania, supporting our statement that some morphological differences were apparent, by removing ontogenetic confounding and suggesting overall shape differences. We now note this and that all measured individuals were adults.

Unfortunately, not all specimens were sexed so we comment on this too. We were able to determine sex for over half the specimens (29 individuals out of total 45 measured), so could not fully analyse sex differences in morphology, but argue against that different sex ratios at each location are driving differences in shape.

We included Tasmanian samples (n=5 only) separately in biplots and both LDA and PCA plots (see above).

Data availability:

Information on sequencing statistics and genotyping metrics for each sample need to be given. Please give the vcf or genepop files and fasta files of loci should be deposited in Data Dryad or uploaded as Supplementary Material if file size allows.

**Response**: We have added # of reads and % missing loci for each individual to S1 Table. Also, we have now uploaded a vcf and genepop file as S1 Data and S2 Data as supporting data files. We added following sentence:

“Fastq sequence files were deposited in NCBI’s Sequence Read Archive (accession numbers: SRR12395874-12395945) and the associated metadata is available at GEOME [36] and is provided in S1 Table. Data generated as vcf file (S1 Data) and as genepop file (S2 Data) are available as supporting information.”

Minor comments:

L.1 Title: I personally also like “weedy” seadragon too but common seadragon should be used to support the attempt to standardize names <https://www.fishnames.com.au/>.

**Response:** We agree that common seadragon is proper, however these animals in particular are known widely as ‘weedy’ and is used generally in the literature (see ref’s 23-25, 28 and 52), as well as the IUCN Red List.

We have added “common” to the title as well as at the start of the Abstract, and do say that the proper name is common, see also our response to Reviewer 1.

L.4 Rephrase to “Population genomics of the weedy seadragon” would be more appropriate given the title

**Response**: Changed as suggested.

L.23 The IUCN citation (?) seems misplaced here.

**Response**: Removed whole sentence.

L.23 Unclear, range wide genetic population data is available in Wilson, Stiller & Rouse 2017.

**Response**: Removed that sentence see above.

L.28 replace [] with ()

**Response**: Corrected

L.29 It is unclear how IBD can support the existence of 4 distinct genetic clusters, because IBD measures the degree of increasing genetic differentiation with geographic distance and does not delineate clusters

**Response**: We removed sentence. Instead related our reults of genetic clusters to DAPC and Bayesian clustering analysis.

L.54 Spell out scientific name Syngnathidae first and then refer with the common name in the second sentence

**Response**: Corrected

L.65 missing )

**Response**: Corrected

L.74 Please cite the version of the IUCN assessment because the status of common seadragons has changed throughout different assessments. The IUCN status (now reversed to Least Concern) should also be given.

**Response**: Added sentence: “The weedy seadragon’s status on the IUCN Red List was changed in 2017 from “Near Threatened” (NT) to “Least Concern” (LC) but their population trend is still considered as “Decreasing”

(<https://dx.doi.org/10.2305/IUCN.UK.2017-2.RLTS.T17177A67624517.en>).”

L.84 There is controversy in the exact age of the Last Glacial Maximum but 20-25 KY seems to old. Lewis, S. E., Sloss, C. R., Murray-Wallace, C. V., Woodroffe, C. D., & Smithers, S. G. (2013). Post-glacial sea-level changes around the Australian margin: a review. Quaternary Science Reviews, 74, 115–138.

**Response**: Thank you for the REF which we added now. We would like to add that this particular paper concentrated on the post-glacial sea level fluctuations and does mention several times that the exact LGM times for Tasmania/Vic are controversial.

According to Lambeck and Chappell 2001 as you also mentioned below (added this REF as well), the peak of LGM was around 25kya.

We based our dates on the isthmus (land bridge) that existed when VIC and TAS were connected (thus separating marine organisms between east and west coast) see also Lambeck and Chappell 2001. The opening of land bridge to the east was about 14 kya suggested by Lambeck and Chapell’s 2001 Fig 4.

L.100 Reference to Supp Table 2 with collection needed

**Response**: Added. “In total 82 *P. taeniolatus* were sampled for genetic analysis between astral summer 2014/15 – 2018 (S1 Table).”

L.107 Please specify how many individuals were measured from each locality.

**Response**: Added sentence and provide full data now in S2 Table (morphology).

“In total we measured 45 individuals, 20 each from NSW and VIC and 5 from TAS (S2 Table).”

L.123 What barcodes were used? Were they single or dual barcodes?

**Response**: Sentence added. “The RADSeq data generated by AGRF used single barcodes that are incorporated into the names of the Fastq files for each individual.”

L.140 Please provide some detail of how STACKS calls SNPs as this is an important factor determining the number and quality of the raw SNPs. How was the single SNP chosen, randomly or according to best depth of coverage?

**Response**: We have added a reference to the manuscript Rochette et al. (2019) that explains in section 2.8 the Bayesian genotype caller employed by STACKs V2 to call SNPs. We used the ‘*write_single_snp*’ option which outputs the first SNP per locus and the default setting which for min coverage with is 3x per individual which resulted in a mean coverage of 21.1 x per individual per locus. See text below.

The manuscript now says:

“Demultiplexing of the sequence data and initial quality control was conducted at AGRF with STACKS v.1.41 [36,37] using default settings. Only sequences with exact barcodes were retained and sequences were trimmed to 140 bp. All other data processing took place at the Genomics and Bioinformatics Cluster at the University of Central Florida using STACKS v.2.0b. Loci were assembled using the denovo pipeline using default settings except we allowed for four mismatches between stacks within individuals (*-M*) and four mismatches between sample loci when building the catalog (-n). See Rochette et al. [39] for a detailed explanation of the Bayesian genotype caller employed in STACKS.

Stacks in which the numbers of reads were more than two standard deviations above the mean were assumed to be repetitive elements and removed from the data set. To eliminate poor quality samples, those individuals with fewer than 200,000 reads or less than 60% of loci were excluded from further analysis, resulting in a final sample size of 72 individuals. Using the ‘*populations*’ function in STACKS, we eliminated those loci with observed heterozygosity greater than 0.7 (--max-obs-het) and applied a minor allele frequency cut-off of 0.05 (--min-maf). We retained only those loci that amplified in ≥ 80% of individuals (-r). Output files were created by implementing the ‘*write_single_snp*’ option which outputs the first SNP per locus.”

And the results read:

“A total of 19,608 loci were resolved across 72 individuals of which 7462 were variable and passed filtering. Samples had an average coverage 21 reads per SNP with a data matrix that was 92.6% complete (S1 Table for % missing loci per individual).”

Please see comments below for more information by author of STACKs.

L.142 What was the requirement for depth of coverage?

**Response**: The default settings for STACKs is 3x. We used the default which results in an average coverage of 21x per individual per locus. This decision was made after a conversation with the authors of STACKs Julian Catchen.

From the author of Stacks: “There is no minimum depth to call SNPs. This has always been handled by
the SNP model, which calculates the likelihood of each position being a
heterozygote and a homozygote (while incorporating a prior reflecting
the allele frequencies in the wider populations). The model then weighs
the evidence for a heterozygous versus a homozygous position using a
likelihood ratio test resulting in a p-value.

If you want to require more evidence, that is more reads, to support a
particular SNP call, you can increase the required p-value to consider
the likelihood ratio test statistically significant (--alpha flag to
gstacks). The default p-value is (of course) 0.05, but you can lower
this, which will require more evidence for a call to be declared
statistically significant.

Using -m at the population-program stage to randomly drop alleles that
have already proven to be statistically significant is a bad idea and
will result in random allele drop out. I regret ever making this option
available and removed it when given the chance with the 2.0 release.”

<https://groups.google.com/forum/#!topic/stacks-users/IzeSy3nw5Bg>

L.144 Abbreviations should be defined as observed heterozygosity (Ho) etc.

**Response**: Added

L.152 How many replicates to assess significance?

**Response**: We are unsure where this ‘replicates to assess significance’ is?

Line 151-153 reads:

“…. comparisons according to Benjamini and Yekutieli [37] by controlling false discovery rates using the p.adjust function in R. In addition, we tested for Isolation by Distance [38] using a Mantel test and 100,000 permutations, as implemented in the R package ade4 v.1.7-15 [39].”

As far as we can tell there are no missing # of replicates in this section.

L.159 The sentence seems incomplete.

**Response**: Edited: “To explore the possibility of population structuring, we used two methods, Bayesian clustering algorithms of STRUCTURE and Discriminant Analysis of Principle Components (DAPC).”

L.165 Please also include a simple PCA that does not require setting a value of K. DAPC aims to maximize the distance between K groups and can often appear like heavier structuring than in the data. A simple PCA allows to look at clustering with very little assumptions.

**Response**: We have now included PCAs for our major groups in supplemental materials (S3 Fig). These do indeed show less clustering compared to the DAPC plots. However, multiple lines of evidence support the DAPC clusters including F-statistics and STRUCTURE analyses so the major conclusions of our study have not changed.

L.165 I may be wrong here but DAPC is PCA based which means that it cannot handle missing data. How were missing data points replaced? What K was chosen for DAPC?

**Response**: Agreed. We’ve added the following text.

“Because PCA analyses require no missing data (and DAPC uses PCA) we replaced missing data with the mean allele frequency for each locus using the method *scaleGen* in ADEGENET.”

A k of 4 was chosen for DAPC, see the S1 Fig for the BIC graph used to determine this.

L.174 What dataset was this done on? SNPs only or RAD loci, were invariant RAD loci included?

**Response**: The phylogenetic tree has been removed from the paper as per Reviewer 1.

L.176 How was the substitution model chosen? I assume the analysis was done on the concatenation of all RAD loci without partitioning?

**Response**: The phylogenetic tree has been removed from the paper (see above).

L.185 What was the sample size for VIC and NSW? Common seadragons are sexually dimorphic with deeper bodies in the females - was this accounted for in the analyses?

**Response**: See also Reviewer 1 above, we changed the morphology section. We added sex where it was possible.

L.196 Please provide a table of sequencing and SNP statistics for each individual. The main text also needs statistics on average missing proportions

**Response**: These data have been added to S1 Table and a line in the text has been added, only 7.4% missing. “Samples had an average coverage of 21 reads per SNP with a data matrix that was 92.6% complete (S1 Table for % missing loci per individual).”

L.197 Unclear which groups this FST value is comparing.

**Response**: Added to new S3 Table A (AMOVA results).

L.206 Please add the population description as in Table 3 to Table 2.

**Response**: Table 3 (morphology students t-test) has been removed and instead we have plotted all measurements against total length to eliminate allopatric patterns.

L.223 Is allelic richness useful for binary SNP markers?

**Response**: We used this so other studies can compare. Some studies use Ho and/or He but a few terrestrial studies use allelic richness. During the course of writing the MS and comparing to other studies using SNPs (mainly terrestrial studies like cattle, horse etc) most authors used allelic richness more often than He or Ho. We attempted to show allelic richness of seadragons for future comparisons to other organisms.

L.233 I think it would be more logical to place the Mantel test directly after the FST.

**Response**: Edited result section moved Mantel test after pairwise Fst.

L.233 and following Please use past tense to describe results.

**Response**: corrected

L.237 DAPC typo

**Response**: corrected

L.241 The interpretation of a monophyletic Eden population and its relationship to the NSW populations depends on the rooting. This needs to be reformulated. It is also not clear what “instead of its own lineage” means. Eden is its own lineage.

**Response**: Removed this sentence as we removed the phylogenetic tree as per Reviewer 1.

L.245 Given the concerns regarding the proposed species complex, “gene flow” would be the better term to use over “hybridisation”.

**Response**: Removed this sentence as per Reviewer 1 and in discussion we edited to ‘interbreeding’.

L.260 Figure legend should mention that only branches with 100 bootstrap support are annotated.

**Response**: Figure 5 was removed as per Reviewer 1

L.265 We would expect allometric scaling of most of these measurements. Where the measurements scaled by the total TL?

**Response**: Added S4 Fig plotting all 15 measurements against total length. In discussion:

“While individuals from Victoria were somewhat smaller, biplots (S4 Fig) indicate that shaped differences summarised in Fig 5A were not simply length-related. Most NSW individuals were not sexed but it is unlikely skewed sex ratios of samples by location can account for shape differences.”

L.291 The isthmus opened later, detailed reconstructions and dates are in Lambeck, K., & Chappell, J. (2001). Sea level change through the last glacial cycle. Science, 292(5517), 679–686.

**Response**: Added citation Lambeck & Chappel clearly state that the land bridge was closed effectively until ca. 14kya but started to break up from 14kya albeit fluctuating.

L.292 Gene flow is more appropriate in this context.

**Response**: Corrected

L.293 The FST values are large but they do not indicate complete isolation.

**Response**: For SNP data there are indeed very large. See more of our explanation above for Reviewer 1. We have edited the sentence and also added Wilson et al 2017.

L.303 Please give the source of this information.

**Response**: we removed colour patterns as suggested by Reviewer 1.

L.313 distant?

**Response**: Corrected

L.313 is almost identical to L296 and following without providing new evidence

**Response**: Edited, this whole section has been re written see p.14 in our MS.

L.329 write out microsatellites

**Response**: Corrected

L.328 This sentence is hard to follow.

**Response**: Edited: “This low genetic diversity in both the endemic weedy (this study) and leafy [34] seadragons stands in comparison with other members of the family Sygnathidae, which have moderate to high heterozygosity. Specifically, H_e_ ranged from 0.452 – 0.949 in five studies of widespread seahorses (*Hippocampus* sp.; [56–59]) and from 0.752 – 0.958 in four widespread pipefishes (*Syngnathus* sp.; [60–63]).”

Figure 1 Sydney is all CAPS, other localities not. Would be helpful to add sample size to the map.

**Response**: Corrected, and sample sizes for each population have been added to legend of Fig 1.

Suppl Table 3 Some measurements have more then 10 decimal places which cannot possibly be the accuracy of the ImageJ measurements

**Response**: Corrected this table is now S2 Table.
