# Supplementary material for: Genomic and morphological evidence of distinct populations in the endemic common (weedy) seadragon Phyllopteryx taeniolatus (Syngnathidae) along the east coast of Australia
Source: PLoS One. 2020 Dec 23;15(12):e0243446. doi: 10.1371/journal.pone.0243446 (PMC7757807; doi:10.1371/journal.pone.0243446)
Supplement: Attachment — Submitted filename: 2nd Response to Reviewer.docx [file pone.0243446.s012.docx]

Response to Reviewer Comments:

Klanten et al. have done a careful job at addressing the reviewers’ concerns and have added

useful figures and additional material that improve the manuscript. My remaining concerns are

regarding the still strong emphasis on a species complex, for which I do not see sufficient

evidence. There are also questions regarding the PCA, which I think is a simple plotting error.

1) Both reviewer 1 and myself have raised concerns about the suggestion of a species

complex in Victoria and New South Wales. The authors have made modifications in

calling for extended sampling and the improved resolution that nuclear markers can give,

which are useful additions. However, the text still strongly implies that there is a species

complex but that separate species are “not yet advocated”. The presented evidence

based on allele frequency differences analyzed in a number of ways is not strong

enough to draw that conclusion. This needs justification from the literature. As I have

pointed out in my last review, this view is omitting the results from Structure at K=2 that

does show shared ancestry components between Victoria and the eastern sites. If there

was reproductive isolation, one would expect completely separate ancestry proportions.

The Structure results need to be mentioned.

**Response**: Structure Harvester analyses indicate the most likely K is four. This is supported by DAPC and in part by AMOVA. We added the following text “Each population harboured private alleles including CNSW (1721), SNSW (348), TAS (563), and VIC (3918). While none of these were fixed a substantial proportion reached an allele frequency of 0.9 meaning they were nearly fixed (CNSW: 1200, 70%; SNSW: 128, 37%; TAS: 161, 29%; VIC: 1385, 35%).” The pattern presented in the Structure plots when looking at all values of K can be explained by incomplete lineage sorting within recently diverged lineages. Further, we would not expect completely separate ancestry proportions, as it takes time for such differences to fix, and these were likely in full contact during the last interglacial period (about 120 kya), and intermittently until the glacial maximum about 40 kya. Finally, due to our results and west to east current of the Bass Strait, there may be occasional unidirectional introgression of Victorian genes to the eastern populations, but the reverse is highly unlikely, which is why we specifically noted this population even though the others also demonstrate high differentiation.

We agree with the Reviewer that more evidence is still needed to confirm distinct species but note that the genetic data, backed up by morphological, are entirely consistent, with the hypothesis that these are diverging populations that are not randomly mating and possibly not mating at all. These, we believe are important considerations for conservation.

We feel that it would be remiss to ignore a reasonable scientific discussion of our results and their interpretation, which we strongly feel should include a hypothesis of a subspecies and how we might further test this. Apart from the evolutionary and ecological implications, conservation management of this iconic species will depend on species population structure across its range.

Therefore, our Discussion includes such speculation and a way forward. However, note that we do not use these terms (subspecies) in our Title, Abstract, and Results.

We would also like to draw the Reviewers attention to a few recent articles that support genome level studies to delineate species-level structure (subspecies, distinct lineages etc) examples are:

1. Baumsteiger et al. 2017, Genomics clarifies taxonomic boundaries in a difficult species complex. PLoS ONE 12(12): e0189417. https://doi.org/10.1371/journal.pone.0189417.

2. Roux et al. 2016, Shedding Light on the Grey Zone of Speciation along a Continuum of Genomic Divergence. PLoS Biol 14(12): e2000234. doi:10.1371/journal.pbio.2000234.

3. Beheregaray et al. 2017, Genome-wide data delimits multiple climate-determined species ranges

in a widespread Australian fish, the golden perch (Macquaria ambigua) Mol. Phylog. Evol 111, 65-75.

2) Along the same vein, there are also multiple mentions of “very restricted to no gene flow”

or similar. This lies on the assumption that FST values reflect migration rates. In

practice, this relationship is not well established because there are also other factors

which impact allele frequency differences (see for example McCauley DE, Whitlock MC.

“Indirect measures of gene flow and migration: FST ≠ 1/(4Nm + 1)” Heredity .

1999;82:117–125). It would be more appropriate to refer to “significant allele frequency

differences” or “significant genetic differentiation”, because this is what has actually been

measured with FST.

**Response**: McCauley and Whitlock’s argument is that one should not directly measure gene flow using Wright’s F-statistics, and we do not do so in this paper. We accept that such a calculation would be inaccurate, however, references to the direction and loose magnitude of link between gene flow and genetic differentiation are still valid. We have altered much of the wording to reduce confusion but have retained it where appropriate.

3) I thank the authors for including the PCA analysis. I think there may have been a mistake

in the PCA plotting. I wondered how such clear clustering in DAPC can result in the PCA

plot as shown in Figure S3, where several individuals were not clustering with their

parent population. If that was true this would be concerning because PCA and DAPC are

conceptually very similar. I repeated the analysis (on genepop file with four population

groups separated) and found a clear assignment of individuals to populations (see code

and plot below). The structure of the PCA looks similar to Figure S3 but it looks like the

coloring of the individuals according to their populations is off in Figure S3. There must

be a glitch in the R script that the authors used. Good news is that the PCA will support

the DAPC results much better and the authors could consider to include it in the main

text (up to the authors).

(The screenshots will likely not appear in the online form, so please see the attached

PDF version of the reviewer comments)

**Response:** We thank Reviewer 2 for catching this! We corrected our code (apparently an older table was used where some of the samples were incorrectly coded).

Minor comments

L.67 Not sure if reference 19 is appropriate for the number of Syngnathidae species;

Reference 18 Eschmeyer actually lists 321 valid species

**Response**: Corrected and removed REF 19

L.129ff. May be more readable as complete sentences?

**Response:** Edited

L.219 Typo in “Principal components”

**Response:** Corrected

L.230 Typo in “patterns”

**Response:** Corrected

L.231 Population code “SYD” has not been defined before.

**Response:** Corrected to NSW (typo as many samples measured were from Sydney area)

L.258 Unclear what the difference between FST = 0.561 and FST = 0.630 (L.262) is.

**Response:** We clarified the difference between the two Fst values. One was for all 9 pops (overall), the second Fst was for grouping the 9 pop’s into 4 areas.

L.289 Move “(FST ranging 0.636– 0.703)” to the end of the sentence to include TAS

**Respose:** Moved to the end of sentence

L.313 Reference to Supplementary Figure S1 needed.

**Response:** Added

L.370 Past tense? “were highly distinct”

**Response:** Corrected

L.373 Move references to after “as a barrier to marine dispersal for 20-25 kya 374 during the last glacial maximum (LGM)”

**Response:** Moved reference

L.376 The strait opened 14 ka ago according to Lambeck & Chappell, so gene flow may

have been reinstated between 14 kya and today, not 14-25 kya.

**Response:** Corrected

L.379 Type in “shape differences”

**Response:** Corrected

L.380 It does not seem obvious why sex differences would not affect the observed

differences.

**Response:** We clarified this by adding a sentence in the Results (most NSW samples were not sexed, and we had only 4 females in VIC see also S2 Table) and explained why we cannot report a skewed sex ratio in the discussion (we had only 10 females out of 45 specimens). They did not influence our linear trendline for body depth, however to resolve this we would need to measure more females especially in NSW where females can be easily distinguished in situ by body depth.

L.383 It is more appropriate to refer to allele frequency differences than gene flow (see

above).

**Response**: We thank the reviewer but can’t find a reference to gene flow on line 383.

L.386 Need a reference that the found differentiation is extraordinarily high.

**Response**: Added Ref 54.

L.388 This indicates that some of the RAD loci obtained the mitochondrial genome. Is

this likely? Intuitively, I would think that is unlikely to obtain mitochondrial RAD loci but maybe

this is different in this case. If there were mitochondrial loci in the dataset, these would have to

get filtered or modeled separately from nuclear loci because they are haploid and have a

different effective population size.

**Response**: We have removed the references to this. We do believe some mitochondrial loci were sequenced, but haploid loci would have been filtered out when we only retained polymorphic sites.

L.398 This paragraph seems isolated and the point is not clear.

**Response**: We moved this sentence up, to where we talk about isolation distinguishing populations

L.405 Unclear which populations “this division” refers to. The previous sentence states

that TAS and NSW populations were historically connected, not divided. The following sentence

also talks about similarities, rather than divisions.

**Response**: We edited and moved this sentence up where we talk about Vic pop managed separately to NSW, TAS pop’s.

L.415 change to “(see also [30])”?

**Response**: Added now it’s REF 29

L.435 Reference needed: “less resilient under normal circumstances, but instead have

a smaller gene pool due to smaller population sizes and restricted distribution ranges”

**Response**: Added references [9, 13]

L.436 It is more appropriate to refer to allele frequency differences than gene flow (see

above).

**Response**: Edited to genetic differentiation

L.440 Reference needed: “increase in anthropogenic stressors resulting in

habitat loss (kelp and seagrass) and an increase of sea surface temperature”

**Response**: Added references

L.458 It is more appropriate to refer to allele frequency differences than gene flow (see

above)._

**Response**: We would like to keep ‘restricted gene flow’ as this section is in general terms and appropriate as it concerns management perspectives.
